# A Double‐Blind Randomised Clinical Trial of Terbinafine‐Nanostructured Lipid Carriers: Should We Anticipate This Strategy for Effective Topical Treatment of Onychomycosis?

**DOI:** 10.1111/myc.70076

**Published:** 2025-06-17

**Authors:** Shima Parsay, Majid Saeedi, Mahdi Abastabar, Mohammad Taghi Hedayati, Seyyed Mobin Rahimnia, Nasim Gholizadeh, Armaghan Kazeminejad, Katayoun Morteza‐Semnani, Roozbeh Zare Gashti, Kofi Asare‐Addo, Maryam Moazeni, Ali Nokhodchi

**Affiliations:** ^1^ Student Research Committee Mazandaran University of Medical Sciences Sari Iran; ^2^ Department of Pharmaceutics, Faculty of Pharmacy Mazandaran University of Medical Sciences Sari Iran; ^3^ Pharmaceutical Sciences Research Centre Haemoglobinopathy Institute, Mazandaran University of Medical Sciences Sari Iran; ^4^ Department of Medical Mycology, School of Medicine Mazandaran University of Medical Sciences Sari Iran; ^5^ Invasive Fungi Research Center Communicable Diseases Institute, Mazandaran University of Medical Sciences Sari Iran; ^6^ Department of Dermatology, Faculty of Medicine Mazandaran University of Medical Sciences Sari Iran; ^7^ Department of Medicinal Chemistry, Faculty of Pharmacy Mazandaran University of Medical Sciences Sari Iran; ^8^ Department of Pharmacology and Toxicology, Faculty of Pharmacy Mazandaran University of Medical Sciences Sari Iran; ^9^ Department of Pharmacy University of Huddersfield Queensgate, Huddersfield UK; ^10^ School of Life Sciences University of Sussex Brighton UK

**Keywords:** antifungal susceptibility test, clinical trial, mycological perspective, NLCs, onychomycosis, severity index, TBF‐NLC 1% gel, terbinafine

## Abstract

**Background:**

Oral terbinafine (TBF) is the drug of choice for onychomycosis management. To treat and heal the rough and thick nail tissue affected by fungal agents, a high dose and plasma concentration of this drug is necessary. This, however, poses a life‐threatening risk due to the cytotoxic side effects, drug–drug interactions, and adverse physical and chemical properties associated with oral medications.

**Objectives:**

This study aimed to employ nanostructured lipid carriers (NLCs) in a gel formulation to avoid side effects and to increase the absorption of topical TBF.

**Methods:**

Terbinafine‐loaded nanostructured lipid carriers (TBF‐NLCs) were developed and optimised using an ultrasonic probe technique, resulting in the formulation of TBF‐NLCs as a 1% w/w carbopol gel after verifying the characteristics associated with NLCs. In vitro antifungal susceptibility test (AFST) was conducted on 85 prevalent fungal species associated with onychomycosis, as well as on strains isolated from trial participants, following the CLSI M38‐A2 and M27‐A3 guidelines. A total of 60 volunteers were enrolled in this clinical randomised, double‐blind, placebo‐controlled study, divided equally into three groups prescribed with TBF cream 1%, TBF‐NLCs gel 1%, and a placebo.

**Results:**

A monodisperse suspension of spherical nanoparticles was successfully produced, exhibiting a zeta potential of 18.4 ± 1.02 mV, a Z‐average of 131.7 ± 5.32 nm, a PDI index of 0.280 ± 0.017, and an EE percentage of 83.51 ± 3.52, all without any cytotoxic effects. The severity index showed a reduction from 65% and 55% to 35% and 10% in the TBF cream 1% and TBF‐NLCs groups, respectively. From a mycological perspective, no significant negative results were noted during the 6th and 8th weeks of TBF‐NLC 1% gel application.

**Conclusion:**

The application of TBF‐NLCs gel 1% demonstrated a quicker clinical recovery without adverse side effects compared to TBF cream, thus highlighting the effective nature of NLCs.

## 1 Introduction

In recent decades, skin and nail fungal infections have emerged as a global concern [1, 2]. These diseases disrupt an individual's quality of life, leading to health and beauty issues, mental disorders, and stress. Additionally, the rise of treatment‐resistant fungal species presents a significant challenge in the realm of healthcare [3]. The initial and gold standard oral treatment for onychomycosis (ONM), which effectively reduces the complications of the disease, is terbinafine (TBF). TBF boasts a mycological treatment rate of 70%–79% and a complete treatment rate of 38%–59% [4]. Besides addressing nail fungal disorders, this medication is also effective against skin infections and pityriasis versicolor [5]. To treat and heal the rough and thick nail tissue affected by fungal agents, a high dose and plasma concentration of this drug is necessary. This, however, poses a life‐threatening risk due to the cytotoxic side effects, drug–drug interactions, and adverse physical and chemical properties associated with oral medications [6, 7]. The rates of treatment failure, relapse, and resistance have escalated to epidemic levels, as evidenced by studies conducted in India, with other cases reported in Asia, Europe, and North America. Resistance primarily arises from mutations in the squalene epoxidase gene, the main target of TBF [8, 9, 10, 11]. While local treatments could potentially replace the oral form, they do not present cytotoxic issues; however, due to their low penetration and release in the hard tissue of the nail, they are not a practical treatment option, as less than 5% of TBF is absorbed in standard topical formulations [12]. Furthermore, this form of medication necessitates a lengthy treatment duration of 6–18 months. Many patients, therefore, tend to discontinue use at the first signs of recovery, before the infection is fully eradicated and complete recovery is achieved [13].

One alternative approach to address these issues is to utilise nanocarriers, in situ gels, and microemulsions for enhanced drug delivery [14]. In addition to resolving numerous challenges related to drug delivery and release, as well as reducing toxicity and costs, this technology exhibits remarkable efficacy against biofilms formed by microorganisms, which serve as a significant defence barrier and contribute to drug resistance [15]. Among the various drug delivery systems, nanostructured lipid carriers (NLCs) and solid lipid nanoparticles (SLNs) are extensively employed for local drug delivery due to their interaction with the lipid membrane through a lipid matrix, thereby facilitating drug delivery [16, 17, 18]. Furthermore, studies have demonstrated that both NLCs and SLNs enhance the hydration of the nail plate, thus promoting deeper drug penetration into the nail. This hydration process increases both the number and size of the pores in the nail plate, thereby facilitating drug absorption into the nail bed [19]. Conversely, it is important to note that NLCs have been developed more extensively than SLNs over the past few decades, with reported greater loading capacity, improved physical stability, and enhanced bioavailability [20].

In this study, the authors aimed to load NLC nanoparticles with TBF (TBF‐NLCs) to achieve a more effective local transfer of TBF into the nail tissue. A double‐blind randomised clinical trial was conducted to determine the appropriateness of TBF‐NLCs as an effective treatment response in the management of ONM.

## 2 Materials and Methods

### 2.1 Materials

Terbinafine (TBF, pharmaceutical grade) was sourced from Fanavarn Daroui Hakim (FDH, Tehran, Iran). Sabouraud dextrose agar (SDA), glyceryl monostearate (GMS), oleic acid, Tween 80, Span 80, dimethyl sulfoxide (DMSO), HPLC‐grade acetonitrile, and methanol were obtained from Merck Co. (Germany). Roswell Park Memorial Institute (RPMI) medium was purchased from Gibco (Thermo Fisher, USA). Morpholinepropanesulfonic acid (MOPS) and 3‐(4,5‐dimethylthiazol‐2‐yl)‐2,5‐diphenyltetrazolium bromide (MTT) solution were acquired from Sigma Chemical Co. (St. Louis, MO, USA). Deionised water was purified using a Milli‐Q system (Millipore, Direct‐Q).

### 2.2 TBF‐Loaded Nanostructured Lipid Carrier (TBF‐NLCs) Preparation

This study employed an ultrasonication method for the preparation of TBF‐NLCs. The methodology was adapted from a previously published study [21]. To prepare TBF‐NLCs, 1% w/w TBF, a solid lipid (0.75% w/w stearic acid) in combination with a liquid lipid (0.25% w/w oleic acid) and a mixture of non‐ionic surfactants (1% w/w Span 80 and 1% w/w Tween 80) were melted at 80°C. The aqueous phase, which consisted of 10 mL deionised water, was heated to the same temperature and poured into the organic phase in a dropwise manner. This was then sonicated using a probe sonicator (Bandelin Sonopuls, Berlin, Germany) for 5 min with 40% amplitude to form a pre‐emulsion. This emulsion was then maintained in an ice bath to form the required NLCs [22]. Together with TBF‐NLCs and carriers without TBF were also prepared as a placebo with the same method described above.

### 2.3 Characterisation of Synthesised TBF‐NLCs

#### 2.3.1 Morphology

TBF‐NLCs were stored at −80°C for 24 h before lyophilization. Lyophilization was performed using an Alfa 1‐2Id Plus freeze dryer (Martin Christ GmbH, Germany) for 24 h. Morphological characterisation of the optimised TBF‐NLCs formulation was conducted using a field emission scanning electron microscopy (FESEM; TESCAN‐MIRA3, Czech Republic). Prior to FESEM analysis, the samples were coated with a thin gold film [23, 24].

#### 2.3.2 Particle Size, Polydispersity Index and Zeta Potential Measurement

Particle size distribution and zeta potential were determined using a dynamic light scattering (DLS) with a Malvern Zetasizer ZS (Nano ZA, Malvern Instruments, UK) instrument. All the samples were diluted 1:5 with deionised water before analysis, and the measurements were performed three times at 25°C with a 90° angle detection. This approach enabled the determination of the particle size distribution and zeta potential of the TBF‐NLCs [25].

#### 2.3.3 Determination of TBF Entrapment Efficiency

To assess the entrapment efficiency (EE %) of the TBF within the NLCs, the concentration of the free and unloaded TBF in the aqueous phase of the TBF‐NLCs suspension was measured. The TBF‐NLCs were subjected to centrifugation at 21,000 rpm for 30 min (Sigma, Germany) and subsequently filtered through a 0.22 μm pore size filter. The concentration of free TBF in the supernatant was then quantified using UV–Vis spectrophotometry at 220 nm. This experiment was performed in triplicate. The EE% was calculated using Equation (1):

(1) $$\mathrm{EE}\%=\left[\left(W_{i}-W_{f}\right)/W_{i}\right]\times 100$$

Where *W*_*i*_ represents the amount of drug initially added to the formulation, and *W*_*f*_ represents the amount of drug in the supernatant.

#### 2.3.4 Differential Scanning Calorimetry (DSC) Analysis

DSC analysis was conducted using a Pyris 6 instrument (PerkinElmer, USA) to characterise TBF, GMS, and the freeze‐dried TBF‐NLCs. For the freeze‐dried TBF‐NLCs, the NLCs were firstly separated through centrifugation and subsequently freeze‐dried using an alpha 1–2 LDplus freeze dryer (Marin Christ, Osterode, Germany) under reduced pressure. Approximately 5 mg of each sample (TBF, GMS, and freeze‐dried TBF‐NLCs) were weighed and sealed in aluminium pans. The sealed samples were then equilibrated at 20°C for 30 min prior to heating the samples to 250°C at a rate of 20°C per min under an inert nitrogen atmosphere [26]. The DSC instrument was calibrated using indium as a standard.

#### 2.3.5 Attenuated Total Reflectance Fourier Transform Infrared (ATR‐FTIR) Study

To investigate the chemical interactions between the components of the TBF‐NLCs formulation, a Cary 630 Fourier Transform Infrared (FTIR) spectroscope equipped with a diamond attenuated total reflectance (ATR) attachment (Agilent Technologies Inc., California, USA) was employed. ATR‐FTIR spectra were acquired for TBF, freeze‐dried TBF‐NLCs powder, GMS, oleic acid, Tween 80, and Span 80 within a spectral range of 4000–400 cm^−1^ with a resolution of 2 cm^−1^ [27]. This analysis provided insights into the potential chemical interactions between the formulation components.

#### 2.3.6 Drug Release Measurement

In vitro release studies were conducted using a dialysis tube technique to evaluate the release profile of TBF from the prepared TBF‐NLCs. A 5 mL aliquot of the initial TBF‐NLCs dispersion was introduced into a dialysis bag with a 12,500 Da molecular weight cut‐off. The bag was then immersed in 600 mL of PBS (pH 6.8) containing 0.1% w/v of tween 80 and maintained at 37.0°C ± 0.1°C and stirred at 100 rpm using a magnetic stirrer. Two milliliters of samples were withdrawn at predetermined time intervals (1, 2, 4, 6, 8, 10, and 24 h) and replaced with fresh simulated intestinal fluid at the same temperature. The withdrawn samples (1.5 mL) were then centrifuged at 25,000 rpm for 30 min, filtered through a 0.22 μm pore size filter, and introduced into the UV–Vis spectrophotometer for analysis at 220 nm [28, 29]. This procedure allowed for the determination of the release profile of TBF from the NLCs over time.

### 2.4 Cytotoxicity Assay

HFF2 cells, derived from human foreskin fibroblasts, were cultured in RPMI 1640 medium supplemented with 2 mM l‐glutamine, 10% (v/v) heat‐inactivated fetal bovine serum (FBS), and 100 IU/mL penicillin with 100 mg/mL streptomycin (Gibco). The cells were maintained at 37°C in a humidified environment containing 5% CO_2_ and 95% air. Each well of a 96‐well plate was seeded with 10,000 HFF2 cells in 100 μL of RPMI medium containing 10% FBS. After a 24‐h adhesion period, a series of doubling dilutions of TBF‐NLCs, placebo, and free drug, ranging from 0.5 to 320 μg/mL, were added to three replicate wells. Cell viability was assessed using the MTT reduction assay. Following a 2‐day incubation, 10 μL of MTT (5 mg/mL stock solution) was added to each well, and the plates were incubated at 37°C for an additional 4 h. The medium was then removed, and the formazan blue formed within the cells was dissolved by adding 200 μL of DMSO. Absorbance was measured at 570 nm using a microplate reader (ELx800, BioTek Instruments, US) [29, 30]. Surveillance was determined using Equation (2).

(2) $$\text{Surveillance}\;\left(\%\right)=\left[\mathrm{OD}570\;\left(\text{sample}\right)/\mathrm{OD}570\;\left(\text{control}\right)\right]*100$$

Where OD570 is defined as optical density.

### 2.5 Preliminary In Vitro Antifungal Susceptibility Testing (AFST) Against TBF‐NLCs

To evaluate the potential activity of TBF‐NLCs, an antifungal susceptibility testing (AFST) was conducted prior to its application in clinical trials. A total of 85 fungal isolates were used for the initial assessment of the antifungal activity of TBF‐NLCs, which included 35, 10, and 40 strains of yeast, dermatophyte fungi, and non‐dermatophyte moulds (NDMs), respectively. The isolates were obtained from patients suffering from superficial fungal infections and were preserved in the reference culture collection of the Invasive Fungi Research Center (IFRC, Sari, Iran). The isolates had been previously identified to the species level through the sequencing of specific genes/regions, including the internal transcribed spacer (ITS1‐5.8s‐ITS2) for yeasts and dermatophytes, and B‐tubulin for NDM species. AFSTs were performed according to the modified M38‐A3 and M60 document for filamentous fungi and yeasts, respectively [31]. Together with TBF‐NLCs, placebo (the vehicle without TBF), and also a reference antifungal (TBF cream 1% (as TBF‐hydrocholoride, Tehran Shimi, Iran)) were utilised. The reference antifungal was selected according to the dermatologist's most prescribed antifungal used to manage ONM. TBF‐NLCs were tested with concentrations ranging from 5000 to 0.001 μg/mL (0.5%–0.0000001%) while the concentration of TBF was selected according to the reference standard (CLSI M38‐A2) from 16 to 0.016 μg/mL. *Paecilomyces variotii* (ATCC 22319) and *Candida parapsilosis* (ATCC 22019) were used as reference strains (quality control). For TBF, the outcome was interpreted visually while for TBF‐NLCs and placebo, results were deciphered with the aid of an inverted microscope due to their turbidity (Motic AE31, Hong Kong, China).

### 2.6 Formulation of TBF‐NLCs Topical Gel

TBF‐NLCs were incorporated into a 1% w/w carbopol gel base containing sodium benzoate 1% w/w as a preservative. The concentration of TBF in the prepared gel was chosen to be 1% to allow for direct comparison with the commercially available TBF ointment/lotion 1% (TBF cream 1% (as TBF‐hydrocholoride, Tehran Shimi, Iran)), which is the standard treatment for ONM. The carbopol gel was allowed to hydrate for 24 h in deionised water. The resulting mixture was then stirred and neutralised with triethanolamine (approximately 10 drops) to achieve an appropriate semi‐solid carbopol gel matrix [32].

### 2.7 Study Design

#### 2.7.1 Ethics Statement

The present investigation was carried out in accordance with the ethical standards established in The Code of Ethics of the World Medical Association (Declaration of Helsinki) pertaining to research involving human participants. The research protocol received endorsement from the Ethics Committee of Mazandaran University of Medical Sciences, identified by the reference number IR.MAZUMS.REC. 1401.14985. Additionally, the study was registered with the Iranian Registry of Clinical Trials (IRCT) under the registration code IRCT20210611051539N4, which is accessible at https://www.irct.ir/. This registration promotes transparency and ensures the availability of trial information. Informed consent was secured from all participants, who were made aware of their right to withdraw from the study at any point without incurring any adverse consequences.

#### 2.7.2 Study Design

A clinical randomised, double‐blind, placebo‐controlled trial was conducted for patients suffering from ONM caused by various etiologic agents. Volunteers with both clinical and mycological diagnoses of ONM were included in the study. All individuals aged 18 years and older with medium to moderate ONM referred by dermatologists were evaluated according to the ONM Severity Index (OSI) [33], mycological criteria, including positive results for the 20% KOH direct examination test and positive culture on SDA medium. The lesion of each volunteer's nail was graded accurately according to published protocols [33]. TBF tablet 250 mg (Lamisil, Tehran Shimi, Iran) was prescribed by dermatologists as the drug of choice for their current ONM treatment. Individuals who had used any antifungals in the last month were excluded from the study. A total of 60 volunteers participated in the present study and were divided into three groups: 20 volunteers received TBF cream 1% (as TBF‐hydrocholoride, Tehran Shimi, Iran) as the topical antifungal available on the market; 20 volunteers received TBF‐NLCs 1% gel; and 20 volunteers received a placebo as topical agents. Each group was then split up into two equal parts: half had ONM affecting their fingernails, while the other half had toenail involvement. The patients were instructed to apply the drug topically onto the nail tissue twice daily—in the morning and evening. Finally, all volunteers were assessed based on clinical as well as mycological criteria, including onycholysis, dystrophy, paronychia, the OSC index [34, 35], KOH‐direct examination, and also culture outputs at 2, 4, 6, and 8 weeks after antifungal prescription. Oral terbinafine, as 250 mg tablets, was prescribed by dermatologists for routing ONM management.

### 2.8 Evaluation of Outcome

After 60 days of application, the effects of topical treatment with TBF cream 1%, placebo, and TBF‐NLCs 1% gel were evaluated based on clinical and mycological criteria. Assessments were conducted before treatment (Day 0) and at intervals of 2 weeks (Day 14), 4 weeks (Day 30), 6 weeks (Day 40), and 8 weeks (Day 60) post‐treatment. Parameters such as the presence or absence of onycholysis, paronychia, inflammation of the surrounding nail tissue, dystrophy, recovery of nail OSC characteristics, and negative results for the KOH direct examination test and culture were recorded and analysed statistically.

### 2.9 Molecular Identification and AFST of Etiologic Agents

The etiologic agents of ONMs were identified using DNA sequencing methods. To achieve this, *TEF1* (translation elongation factor 1 complex), *TUB1* (beta‐tubulin) genes, and ITS rDNA region sequences were amplified for *Fusarium* sp., NDMs, and dermatophyte/yeast species, respectively. The sequences of the primer pairs used for each region's amplification were as follows: EF1‐F (5′‐ATG‐GGT‐AAG‐GAG‐GAC‐AAG‐AC‐3′), EF1‐R (5′‐GGA‐AGT‐ACC‐AGT‐GAT‐CAT‐GTT‐3′), BT1‐F (5′‐AAC‐ATG‐CGT‐GAG‐ATT‐GTA‐AGT‐3′), and BT1‐R (5′‐TAG‐TGA‐CCC‐TTG‐GCC‐CAG‐TTG‐3′), along with ITS 1 (5′‐TCCGTAGGTGAACCTGCGG‐3′) and ITS 4 (5′‐TCCTCCGCTTATTGATATGC‐3′) [36]. Genomic DNA was extracted using a phenol/chloroform extraction protocol, followed by PCR amplification performed on a Corbett Research thermal cycler, model CG1‐96 (Sydney, Australia). The PCR reactions were conducted in a 50 μL volume containing 25 ng of template DNA, 25 pmol of each primer, and 12.5 μL of reaction master mix (Amplicone, The Netherlands). BT2 amplification involved an initial denaturation step at 95°C for 5 min, followed by 35 cycles of 95°C for 45 s, 60°C for 120 s, and 72°C for 60 s, with a final extension at 72°C for 7 min. Amplification of the *EF1* gene and *ITS* region was performed using the same protocol, except for the annealing temperatures, which were set at 47°C and 56°C, respectively. PCR products were sent to Gene Fanavaran Company (Tehran, Iran) for purification and sequencing of the regions with the previously mentioned primers. The sequences of the genes were compared with the published GenBank sequences (http://www.ncbi.nlm.nih.gov). AFST assays were then performed for the isolates collected from ONMs according to the CLSI published documents previously described in section 2.5.

### 2.10 Statistical Analysis

Statistical analysis was conducted using SPSS for Windows (version 22; SPSS Inc., Chicago, IL, USA). Descriptive statistics were used to assess frequency and demographic data. Continuous variables were analysed within groups using the paired *t*‐test and between groups using the independent *t*‐test. Categorical data were compared between groups using the Chi‐square test or Fisher's exact test, as appropriate. For nonparametric paired data, the McNemar test was applied. A *p*‐value of less than 0.05 was considered statistically significant in all analyses.

## 3 Results and Discussion

### 3.1 Characterisation of Synthesised TBF‐NLCs

TBF‐NLCs were prepared using a melt‐dispersion method followed by ultrasonication. The organic phase consisted of TBF, lipid excipients, and the binary mixture of non‐ionic surfactants, while the aqueous phase contained deionised water. The resulting TBF‐NLCs exhibited a pH of 5.6 ± 0.2, appropriate for topical administration [37]. DLS was employed to characterise the TBF‐NLC formulation's mean particle size, polydispersity index (PDI), and zeta potential (Table 1). The results indicated nanoscale dimensions, a uniform particle size distribution, and a favourable zeta potential, all contributing to formulation stability. The encapsulation efficiency (EE) of TBF in the NLCs was approximately 83%.

**TABLE 1:** Properties of TBF‐NLC formulation. Each measurement was performed in triplicate and the results are presented as mean ± SD.

Formulation	Particle size (nm)	PDI	Zeta potential (mv)	EE (%)
TBF‐NLC	131.7 ± 5.32	0.280 ± 0.017	18.4 ± 1.02	83.51 ± 3.52

Literature suggests that particle sizes below 200 nm enhance cellular uptake and penetration [38], while those under 300 nm are desirable for topical drug delivery [39]. The synthesised TBF‐NLCs exhibited a mean particle size of approximately 130 nm, demonstrating optimal size homogeneity and stability, likely attributed to the emulsifying and carrier properties of the incorporated surfactants and lipids. The positive zeta potential of the nanoparticles is possibly due to the presence of surfactants or permeation enhancers [40].

### 3.2 Morphology Study

**FIGURE 1:**
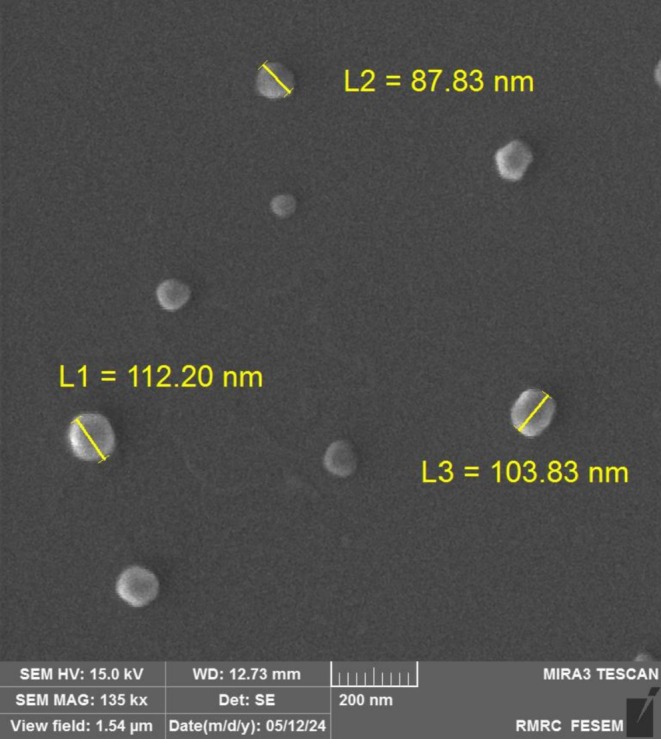
The FESEM image of terbinafine‐loaded NLCs (TBF‐NLC) providing direct visualisation of the particle's physical dimensions.

FESEM image (Figure 1) revealed TBF‐NLCs with an almost spherical morphology and uniform shape. These observations align with the particle size data obtained from DLS (Table 1), indicating a consistent size distribution. The observed discrepancies between DLS and FESEM results are likely due to differences in measurement methodologies, sample preparation techniques, inherent instrument limitations, and the intrinsic properties of the nanoparticles themselves. Notably, DLS measures the hydrodynamic diameter, which accounts for the hydration layer surrounding the particle, while FESEM provides direct visualisation of the particle's physical dimensions. This difference in measurement principle can explain the observed variations in size estimations.

### 3.3 ATR‐FTIR Spectra Analysis

**FIGURE 2:**
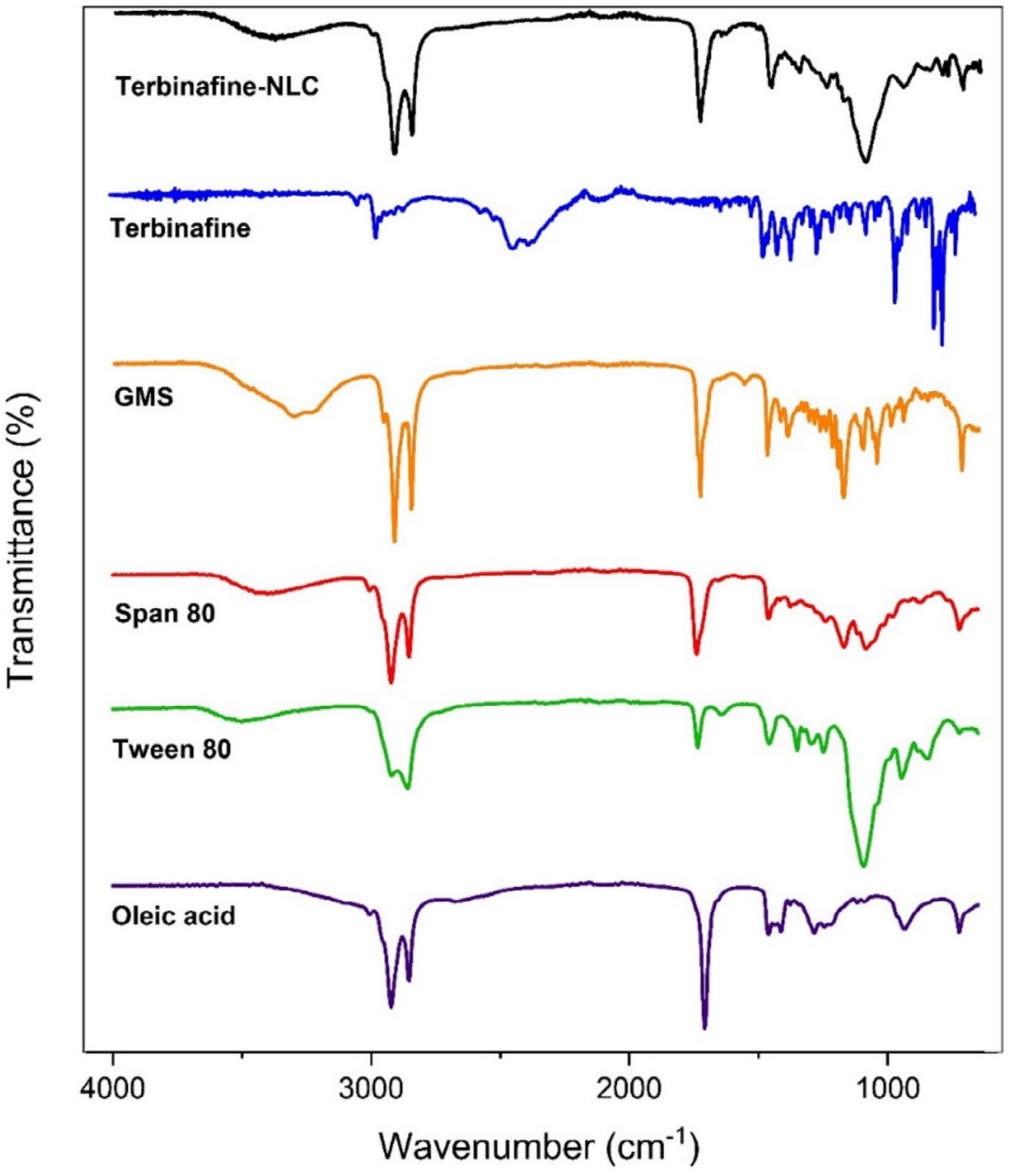
The ATR‐FTIR spectra of the freeze‐dried TBF‐NLC and starting materials terbinafine, GMS, Span 80, Tween 80, and oleic acid.

ATR‐FTIR analysis confirmed the characteristic functional groups of the individual components. TBF exhibited peaks consistent with aromatic C–H stretching (3114, 3075, 3040 cm^−1^), S–H stretching (2518 cm^−1^), C ≡ N stretching (2201 cm^−1^), C=C alkene stretching (1700, 1653, 1638 cm^−1^), C=N stretching (1634 cm^−1^), C=C aromatic stretching (1556 cm^−1^), and C–Cl stretching (1101, 758 cm^−1^) [41]. Oleic acid showed characteristic C–H stretching (2923, 2852 cm^−1^), C=O stretching (1707 cm^−1^), and C–O stretching (1285 cm^−1^) [41], while GMS displayed peaks indicative of O–H stretching (3299–3236 cm^−1^), C–H stretching (2956–2850 cm^−1^), C=O stretching (1729 cm^−1^), and C–O bending (1176 cm^−1^) [42]. The ATR‐FTIR spectrum of Tween 80 demonstrated the main peaks at 3502 cm^−1^ (O–H stretching), 2922 cm^−1^ (C–H asymmetric stretching), 2859 cm^−1^ (C–H symmetric stretching), 1735 cm^−1^ (C = O stretching), and 1093 cm^−1^ (C–O stretching). The ATR‐FTIR spectrum of Span 80 revealed the peaks at 3401 cm^−1^ (O–H stretching), 2923 cm^−1^ (C–H asymmetric stretching), 2854 cm^−1^ (C–H symmetric stretching), and 1739 cm^−1^ (C = O stretching). As shown in Figure 2, the comparative ATR‐FTIR spectrum of the TBF‐NLC formulation revealed no significant shifts in the characteristic peak positions of TBF or the excipients, indicating the absence of chemical interactions between the drug and the excipients.

### 3.4 DSC Thermogram Investigation

**FIGURE 3:**
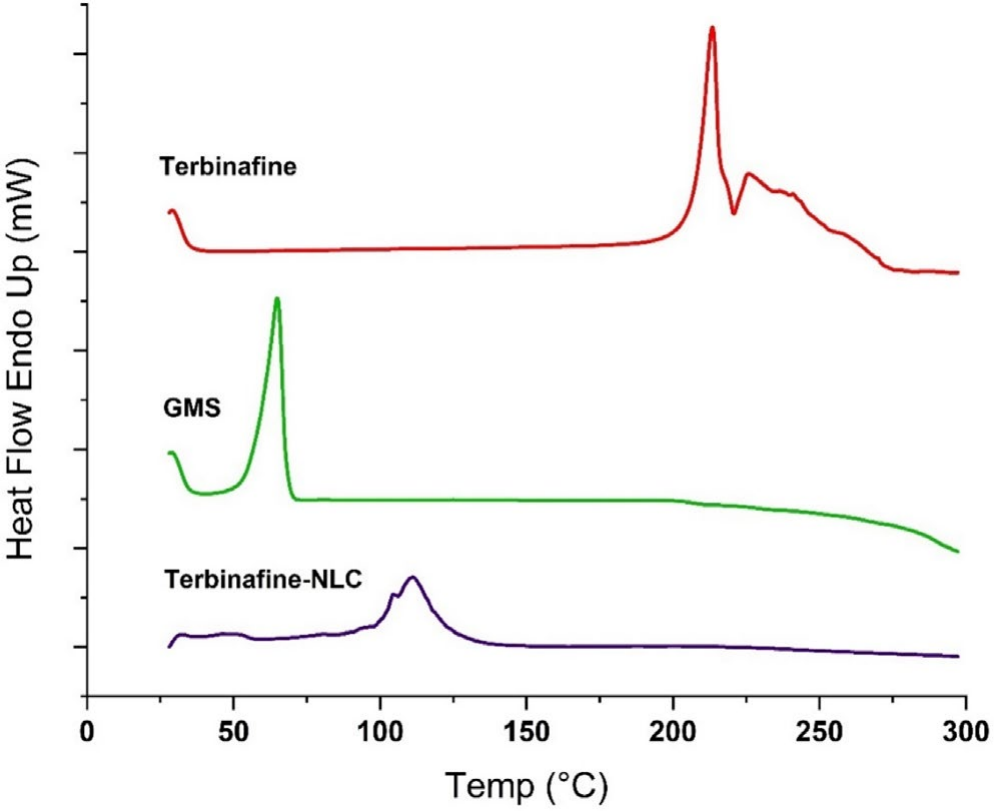
The DSC thermograph of TBF, GMS, and freeze‐dried terbinafine‐NLC.

DSC thermograms (Figure 3) revealed sharp endothermic peaks corresponding to the melting points of pure TBF (~210°C) and pure GMS (~65°C), consistent with literature values [22, 29]. The degradation of TBF occurred around 229°C [42]. Analysis of the TBF‐NLC thermogram revealed a broad, low‐intensity endothermic peak in the range of 100°C–120°C, which is attributed to the desorption of adsorbed moisture from the sample [43]. The absence of these characteristic peaks in the formulation's DSC thermogram indicates the successful incorporation of TBF into the lipid matrix during the preparation of the NLCs and subsequent drug encapsulation.

### 3.5 In Vitro Drug Release

**FIGURE 4:**
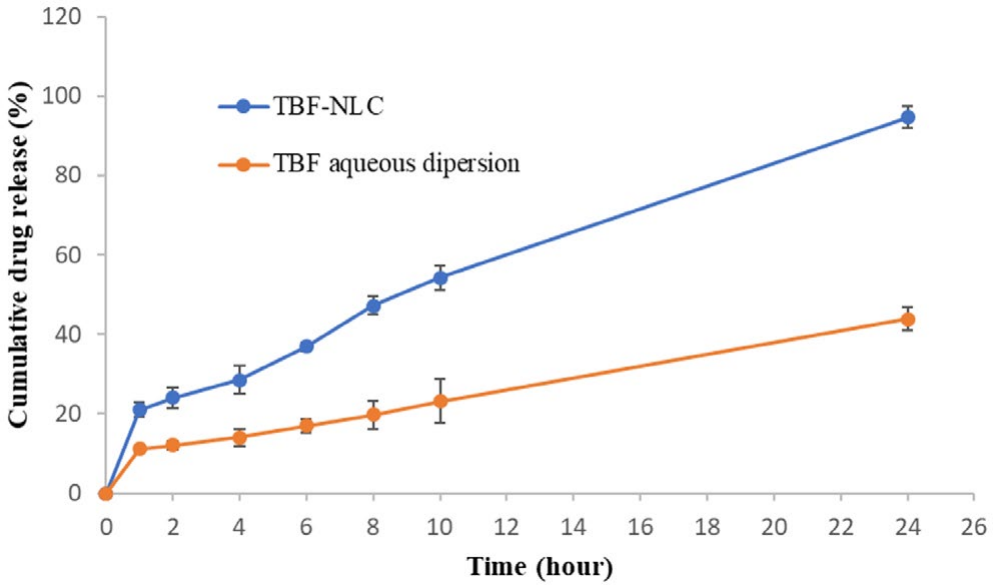
In vitro release profiles of TBF from the NLC vehicle and its aqueous dispersion in PBS (pH = 6.8). The data is presented as mean ± SD (*n* = 3). The difference was significant for the 24 h period (*p* < 0.05).

An in vitro release test was carried out to observe the release behaviour of TBF‐NLC dispersion (1% w/v of TBF) in comparison to TBF aqueous dispersion (1% w/v of TBF in deionised water) (Figure 4). In vitro release studies revealed a biphasic release profile for TBF from the NLCs, characterised by an initial rapid release followed by a sustained release phase. This biphasic release is likely due to the initial release of TBF from the NLC surface, followed by a slower release from the solid lipid core. Compared to the TBF dispersion (43.85% ± 2.57%), the NLC formulation exhibited faster and greater cumulative drug release (94.71% ± 2.87%) after 24 h (*p* < 0.05), potentially due to the presence of surfactants and liquid lipid enhancing drug solubilisation and release [44]. The initial burst release is likely a consequence of drug enrichment in the outer layers of the NLCs during the cooling process [45], whereas the sustained release is attributed to the deep entrapment of TBF within the nanoparticles and the precipitation of oil droplets within the solid lipid matrix following lipid crystallisation, contributing to prolonged release [46].

### 3.6 Cytotoxicity Assay

**FIGURE 5:**
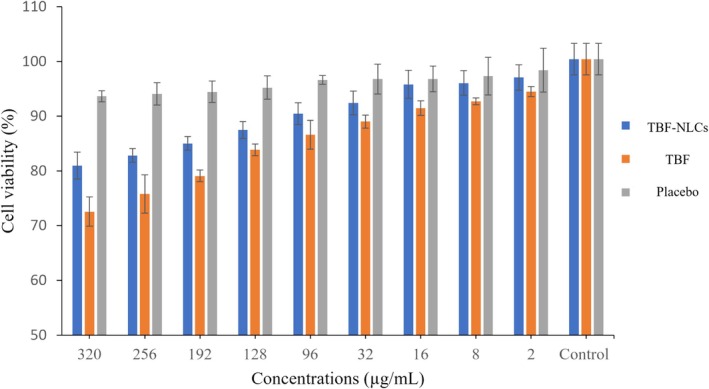
Cell viability rate (HFF cell line) of TBF solution, placebo, and TBF‐NLCs.

MTT assay was employed in this study to assess the cell viability of TBF‐NLCs. Concentrations ranging from 2 to 320 μg/mL of TBF solution, TBF‐NLCs, and the placebo were evaluated. Cell viability significantly decreased after 24 h in both TBF‐NLCs and TBF solution (*p* < 0.05) (Figure 5). The protective effect of TBF‐NLCs was greater than that of the TBF solution. A previous study has shown that NLCs can improve stability and solubility and are non‐cytotoxic, non‐immunogenic, and regarded as safe [47]. Low cytotoxic activity for the vesicular preparations (placebo and TBF‐NLCs) was also noted due to the enzyme's impact on the ester bond [48].

### 3.7 In Vitro Antifungal Susceptibility Testing Against TBF‐NLCs

The AFST results were interpreted and established using CLSI guidelines for NDMs, dermatophytes, and yeasts. Accordingly, conflicting data were observed in MIC values against TBF‐NLCs. The MIC values were not decreased for all three groups of fungi: the TBF‐NLCs were the most effective against NDMs, especially the *Aspergillus* species and also TBF‐susceptible *T. mentagrophytes*. For TBF‐NLCs, the MIC_90_ were achieved as 0.15 μg/mL, 0.08 μg/mL, and 0.08 μg/mL against *A. fumigatus* , *A. flavus* , and *A. niger* , respectively (*p* < 0.05). MIC_90_ values were obtained as 0.25 μg/mL for all three *Aspergillus* species against the TBF solution. In the case of TBF‐resistant *T. mentagrophytes*, yeasts, and *Fusarium* species, elevated MIC values were observed when tested against TBF‐NLCs. Tables 2 and 3 present the detailed results of the AFST assay for 85 fungal isolates. Previous reports have confirmed the effective antifungal results for drug‐loaded NLCs on different fungal species. A notable decrease was observed in MIC values when fluconazole (FLZ)‐loaded NLCs and FLZ‐loaded SLNs formulations were applied [48]. Based on the obtained results, FLZ‐NLCs were significantly more effective (*p* < 0.05) on the most common *Candida* species when compared to FLZ‐SLNs [21]. Motedayen et al. also documented that the nanoparticles of TBF had a great antifungal activity against the dermatophyte isolates, suggesting that it might be used as an alternative agent for dermatophytosis treatment [49]. Drug penetration into the fungal cells is consistently challenging and affects the MIC. Nanoparticles, especially NLCs, are therefore a proposed solution as they facilitate the penetration procedure and release a higher concentration of antifungal agents into the fungal cells [21, 34, 35, 50].

**TABLE 2:** The susceptibility of the onychomycosis‐most related filamentous fungal species against TBF solution and TBF‐NLCs. The geometric mean, MIC_50_, and MIC_90_ values were also determined.

	Isolates	MIC (μg/mL)		Isolates	MIC (μg/mL)		Isolates	MIC (μg/mL)		Isolates	MIC (μg/mL)		Isolates	MIC (μg/mL)	
		TBF	TBF‐NLC		TBF	TBF‐NLC		TBF	TBF‐NLC		TBF	TBF‐NLC		TBF	TBF‐NLC
	*Fusarium* spp. (*n* = 10)	16	2500	*Trichophyton mentagrophytes, TBF‐resistant* (*n* = 7)	16	2500	*Aspergillus fumigatus* (*n* = 10)	0.25	0.15	*Aspergillus flavus* (*n* = 10)	0.25	0.15	*Aspergillus niger* (*n* = 10)	0.25	0.15
		16	2500		16	1250		0.25	0.15		0.25	0.08		0.25	0.08
		16	2500		16	1250		0.125	0.15		0.25	0.08		0.25	0.08
		16	2500		16	1205		0.125	0.15		0.125	0.03		0.125	0.08
		16	2500		16	1250		0.125	0.02		0.125	0.02		0.125	0.08
		16	2500		4	1		0.125	0.02		0.125	0.02		0.125	0.08
		0.5	150		4	1		0.125	0.02		0.0625	0.02		0.125	0.03
		0.5	150		4	1		0.0625	0.02		0.0625	0.02		0.125	0.03
		0.5	150		4	1		0.0625	0.02		0.0625	0.01		0.125	0.03
		0.5	150		2	0.5		0.0625	0.01		0.0625	0.01		0.125	0.01
*MIC values*															
MIC 50		16	2500		4	1		0.125	0.02		0.125	0.02		0.125	0.08
MIC 90		16	2500		16	1250		0.25	0.15		0.25	0.08		0.25	0.08
GM		4	811.33		7.464264	35.35534		0.125	0.0		0.11	0.029265		0.15	0.0

Abbreviations: GM, Geometric mean; MIC, Minimum inhibitory concentration; MIC_50_, minimal concentration that inhibits 50% of isolates; MIC_90_, minimal concentration that inhibits 90% of isolates; TBF, Terbinafine solution.

**TABLE 3:** The susceptibility of the onychomycosis‐most related *Candida* species against TBF solution and TBF‐NLCs. The geometric mean, MIC_50_, and MIC_90_ values were also determined.

	Isolates	MIC (μg/mL)		Isolates	MIC (μg/mL)		Isolates	MIC (μg/mL)	
		TBF	NLC‐TBF		TBF	NLC‐TBF		TBF	NLC‐TBF
	*Candida albicans* (*n* = 15)	16	5000	*Candida glabrata* (*n* = 10)	16	5000	*Candida parapsilosis* (*n* = 10)	16	5000
		16	5000		16	1250		16	2500
		16	5000		16	1250		16	2500
		16	2500		16	1250		16	1250
		16	2500		16	1250		16	1250
		16	2500		16	625		8	1250
		16	2500		16	625		8	1250
		16	625		16	625		8	1250
		16	625		16	312		8	1250
		16	150		16	80		4	80
		16	80						
		16	80						
		16	80						
		16	80						
		16	80						
*MIC values*									
MIC 50		16	625		16	625		8	1250
MIC 90		16	5000		16	1250		16	2500
GM		16	622.9		16	769.2		10.55606	1249.8

Abbreviations: GM, Geometric mean; MIC, Minimum inhibitory concentration; MIC50, minimal concentration that inhibits 50% of isolates; MIC90, minimal concentration that inhibits 90% of isolates; TBF, Terbinafine solution.

### 3.8 Evaluation of Outcome, Molecular Identification and AFST of the Isolated Strains

**FIGURE 6:**
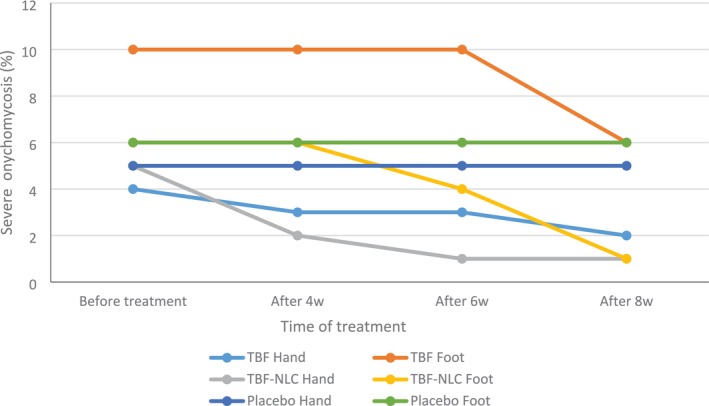
Results for “severity index” after using TBF‐NLCs 1% gel, TBF cream1% as well as placebo in the studied groups.

TBF cream 1%, TBF‐NLCs 1% gel, and placebo were prescribed for patients whose ONM were confirmed according to mycological criteria. The patient's age ranged from 17 to 38 years old, in which the range > 60 years old (33.33%) was affected the most. Women comprised 80% of the affected patients (*n*: 48) of which more than half were housewives (n: 29, 48.33%). As a matter of fact, injured nails are more exposed to fungal infection. Nevertheless, of 60 cases, 37 patients had no trauma or injury to the nail tissue (61.66%). The most prevalent ONM symptom was ONM, which was observed in 52 cases (86.66%) of the patients. Table 4 indicates the detailed demographic data. Regardless of the site of infection (hand or toenail), severe ONM was diagnosed in 58.3% of cases, with the severe form observed in 65% of patients treated with TBF cream 1% (30% for hand nails, 100% for toenails) and 55% of patients treated with TBF‐NLCs and placebo (50% for hand nails, 60% for toenails). After 8 weeks of treatment, the severe cases decreased to 35% and 10% in the TBF cream 1% and TBF‐NLCs groups, respectively. There were no changes in the severity improvement of patients receiving the placebo. This finding strongly indicates the significant effectiveness of TBF‐NLCs on severe ONM, given that the patients in the placebo group were also treated with TBF cream 1%. Detailed information is depicted in Figure 6, which focuses on the “severity index” and also Table 5 (a, b, c), severity index row. In the case of fingernails, 30% of patients experienced a decrease in the severity index after just 4 weeks of TBF‐NLCs application (*p* < 0.05), while this finding was only 10% in patients receiving TBF cream 1%. At the end of the 8‐week treatment period, complete recovery was observed in 30% of patients receiving TBF‐NLCs, compared to 5% of patients receiving TBF and 0% in the placebo group. The clinical manifestations, including paronychia, onycholysis, hyperkeratosis, pain in the surrounding tissue, discoloration, dermatophytoma patch or longitude, were thoroughly examined both before the treatment and also after 2, 4, 6, and 8 weeks of antifungal prescription.

**TABLE 4:** The demographic data of the 60 volunteers who participated in the trial.

	Placebo		TBF‐NLCs		TBF cream 1%		Total (%)	*p*
	Finger nail	Toe nail	Finger nail	Toe nail	Finger nail	Toe nail		
*Age*								
10–20	1	—	—	—	—	—	1 (1.66)	> 0.05
20–30	1	1	2	2	—	—	6 (10)	
30–40	—	3	2	—	3	—	8 (13.33)	
40–50	2	1	2	1	1	2	9 (15)	
50–60	4	3	2	2	2	3	16 (26.66)	
60>	2	2	2	5	4	5	20 (33.33)	
*Occupation*								
Artist	—	—	—	—	1	—	1 (1.66)	ND
Construction worker	—	1	—	—	—	1	2 (3.33)	
Doctor	—	1	—	—	—	—	1 (1.66)	
Driver	—	—	—	1	—	—	1 (1.66)	
Employee	1	—	—	1	1	—	3 (5)	
Engineer	—	—	1	—	—	—	1 (1.66)	
Farmer	1	—	—	1	1	2	5 (8.33)	
Hair styler	1	—	—	—	—	—	1 (1.66)	
Housewife	4	4	7	4	5	5	29 (48.33)	
Laboratory assistant	—	1	1	—	—	—	2 (3.33)	
Nurse	1	—	—	1	1	—	3 (5)	
Retired	1	2	—	—	1	2	6 (10)	
Student	1	1	—	1	—	—	3 (5)	
Teacher	—	—	1	1	—	—	2 (3.33)	
*Gender*								
Male	1	2	—	3	3	3	12 (20)	ND
Female	9	8	10	7	7	7	48 (80)	
*History of trauma*								
Yes	1	7	3	5	—	7	23 (38.33)	
No	9	3	7	5	10	3	37 (61.66)	
Yes	—	—	—	1	—	1	2 (3.33)	
No	10	10	10	9	10	9	58 (96.66)	
*History of underlying diseases, > 0.05*								
Diabetes	1	3	1	2	2	3	19 (31.66)	
Heart disease	2	—	1	2	1	1		
*History of taking antifungal drugs, ND*								
Clotrimazole	1	2	—	2	3	2	14 (23.33)	
Fluconazole	—	—	1	—	—	—		
Terbinafine	—	—	—	—	—	1		
Itraconazole	—	—	1	1	—	—		
*Clinical symptoms*								
Hyperkeratosis	3	2	1	4	—	6	16 (26.66)	
Onycholysis	10	7	9	9	7	10	52 (86.66)	
Pain around the nail	5	—	8	—	8	3	24 (40)	
Paronychia	6	6	6	6	7	5	36 (60)	
Nail discoloration	6	3	2	4	6	7	28 (46.66)	
Dermatophytoma‐patch or longitudinal	4	1	2	3	—	2	12 (20)	
*Microscopic observations*								
Septate/septate branching hyphae	5	7	2	10	1	9	34 (56.66)	
Budding yeast cell	5	3	7	—	8	1	24 (40)	
Hyphae/budding yeast	—	—	1	—	1	—	2 (1.66)	
*Severity*								
Mild	—	2	1	1	—	—	4 (6.6)	
Moderate	5	2	4	3	7	—	21 (35)	
Severe	5	6	5	6	3	10	35 (58.3)	

**TABLE 5:** (a, b, c) The outcome of the clinical trial after using TBF‐NLCs 1% gel (5a), TBF cream 1% (5b) as well as placebo (5c) in the studied groups.

		Before treatment		After treatment							
	Table 5a	TBF‐NLCs		TBF‐NLCs (2 weeks)		TBF‐NLCs (4 weeks)		TBF‐NLCs (6 weeks)		TBF‐NLCs (8 weeks)	
		Finger nail	Toe nail	Finger nail	Toe nail	Finger nail	Toe nail	Finger nail	Toe nail	Finger nail	Toe nail
**Onychomycosis scoring Index**	0							1		5	1
	1			—	—	1	1	3	3	1	3
	2	1	1	1	1	3	3	3	1	3	2
	3	4	3	4	3	4		2	2	—	3
	4	4	3	4	3	2	6	1	4	1	1
	5	1	3	1	3	—		—	—	—	—
	*p*			1.000	1.000	1.000	1.000	0.60	1.000	0.04	0.06
**Severity index**	***p***			**0.25**	**1.000**	**0.008**	**0.021**	**0.004**	**0.004**	**0.004**	**0.004**
**Signs symptoms** ^**a**^	Paronychia	6	6	6	6	5	6	4	4	0	1
	*p*			1.000	1.000	1.000	1.000	0.500	0.500	0.031	0.063
	Onycholysis	9	9	9	9	9	9	5	8	3	5
	*p*			1.000	1.000	1.000	1.000	0.125	1.000	0.031	0.125
	Pain around the nail	8	—	8	—	6	—	2	—	0	—
	*p*			1.000		0.500		0.031		0.008	
	Hyperkeratosis	1	4	1	4	1	4	1	3	1	2
	*p*			1.000	1.000	1.000	1.000	1.000	1.000	1.000	0.500
	Discoloration	2	4	2	4	2	4	1	3	0	2
	*p*			1.000	1.000	1.000	1.000	0.250	1.000	0.125	0.500
	Longitude or patch	2	3	2	3	2	3	2	3	2	3
	*p*			1.000	1.000	1.000	1.000	1.000	1.000	1.000	1.000
**Mycological criteria** ^**c**^	KOH 10% DE	10	10	10	9	10	9	8	8	7	7
	*p*			1.000	1.000	1.000	1.000	0.500	0.500	0.250	0.250
	Culture	10	10	10	7	8	8	7	7	5	5
	*p*			1.000	0.250	0.500	0.500	0.250	0.250	0.063	0.063
**Overall satisfaction** ^**b**^	Dermatologist approval^¥^	10	10	—	1	1	1	3	4	6	5
	*p*				1.000	1.000	1.000	0.250	0.125	0.031	0.063
	Patients’ satisfaction ^£^	10	10	—	2	4	4	9	5	9	8
	*p*				0.500	0.125	0.125	0.004	0.063	0.004	0.008

		Before treatment		After treatment							
	Table 5b	TBF cream 1%		TBF cream 1% (2 weeks)		TBF cream 1% (4 weeks)		TBF cream 1% (6 weeks)		TBF cream 1% (8 weeks)	
		Finger nail	Toe nail	Finger nail	Toe nail	Finger nail	Toe nail	Finger nail	Toe nail	Finger nail	Toe nail
**Onychomycosis scoring Index**	0							—		1	
	1			—	—	—	—	1	—	2	—
	2			—	—	1	—	2	—	2	—
	3	7		7	—	7	—	5	—	4	4
	4	3	4	3	4	2	4	2	4	1	—
	5		6	—	6	—	6	—	6	—	6
	*p*			1.000	1.000	1.000	1.000	1.000	1.000	1.000	1.000
**Severity index**	***p***			1.000	1.000	0.063	1.000	0.031	0.5	0.008	0.125
**Signsx symptoms** ^**a**^	Paronychia	7	5	7	4	7	4	7	3	6	3
	*p*			1.000	0.500	1.000	0.500	1.000	0.125	0.727	0.125
	Onycholysis	7	10	7	10	7	10	6	10	5	10
	*p*			1.000	1.000	1.000	1.000	1.000	1.000	0.500	1.000
	Pain around the nail	8	3	8	3	8	3	7	2	5	1
	*p*			1.000	1.000	1.000	1.000	1.000	0.125	0.500	0.031
	Hyperkeratosis	—	6	—	6	—	6	—	6	—	6
	*p*				1.000		1.000		1.000		1.000
	Discoloration	6	7	6	7	6	7	6	7	6	7
	*p*			1.000	1.000	1.000	1.000	1.000	1.000	1.000	1.000
	Longitude or patch	—	2	—	2	—	2	—	2	—	2
	*p*				1.000		1.000		1.000		1.000
**Mycological criteria** ^**c**^	KOH 10% DE	10	10	10	10	10	8	8	8	7	7
	*p*			1.000	1.000	1.000	0.500	0.500	0.500	0.250	0.250
	Culture	10	10	10	9	8	8	7	7	5	5
	*p*			1.000	1.000	0.500	0.500	0.250	0.250	0.063	0.063
**Overall satisfaction** ^**b**^	Dermatologist approval^¥^	10	10	—	—	1	—	3	—	3	—
	*p*					1.000		0.250		0.250	
	Patients’ satisfaction ^£^	10	10	—	—	2	2	3	3	5	5
	*p*					0.500	0.500	0.250	0.250	0.063	0.063

		Before treatment		After treatment							
	Table 5c	Placebo		Placebo (2 weeks)		Placebo (4 weeks)		Placebo (6 weeks)		Placebo (8 weeks)	
		Finger nail	Toe nail	Finger nail	Toe nail	Finger nail	Toe nail	Finger nail	Toe nail	Finger nail	Toe nail
**Onychomycosis scoring Index**	0										1
	1		1	—	1	—	1	—	2	—	—
	2		1	—	—	—	—	—	—	4	2
	3	5	2	5	3	5	3	5	2	1	1
	4	1	5	1	5	1	5	1	5	2	5
	5	4	1	4	1	4	1	4	1	3	1
	*p*			1.000	1.000	1.000	1.000	1.000	1.000	1.000	1.000
**Severity index**	***p***			1.000	1.000	1.000	1.000	1.000	1.000	0.063	0.25
**Signs symptoms** ^**a**^	Paronychia	6	6	6	6	6	6	6	6	5	5
	*p*			1.000	1.000	1.000	1.000	1.000	1.000	1.000	1.000
	Onycholysis	10	7	10	4	10	3	10	3	10	3
	*p*			1.000	0.250	1.000	0.125	1.000	0.125	1.000	0.125
	Pain around the nail	5	—	5	—	5	—	4	—	3	—
	*p*			1.000		1.000		1.000		0.500	
	Hyperkeratosis	3	2	3	2	3	2	3	2	3	2
	*p*			1.000	1.000	1.000	1.000	1.000	1.000	1.000	1.000
	Discoloration	6	3	6	3	6	3	6	3	6	3
	*p*			1.000	1.000	1.000	1.000	1.000	1.000	1.000	1.000
	Longitude or patch	4	1	4	1	4	1	4	1	4	1
	*p*			1.000	1.000	1.000	1.000	1.000	1.000	1.000	1.000
**Mycological criteria** ^**c**^	KOH 10% DE	10	10	10	9	10	9	10	8	10	7
	*p*			1.000	1.000	1.000	1.000	1.000	0.500	1.000	0.250
	Culture	10	10	10	6	10	5	10	5	6	5
	*p*			1.000	0.125	1.000	0.063	1.000	0.063	0.125	0.063
**Overall satisfaction** ^**b**^	Dermatologist approval^¥^	10	10	—	1	—	1	—	1	—	2
	*p*				1.000		1.000		1.000		0.500
	Patients' satisfaction ^£^	10	10	1	1	1	1	2	3	4	3
	*p*			1.000	1.000	1.000	1.000	0.500	0.250	0.125	0.250

*Note:* A *p*‐value of less than 0.05 was considered statistically significant in all analyses.

^a^ The number of “YES” outcome.

^b^ “Overal satisfaction based on the effect of medication and absence of any side effects” (based on patients' opinion and their satisfaction).

^c^ Mycological criteria was confirmed according to “negative results based on mycologist approval”.

The results obtained suggest ONM, paronychia and nail discoloration were observed in 86.86%, 60%, and 46.66% of volunteers, respectively. When compared to the results for volunteers prescribed TBF cream 1% and placebo, the above signs were totally recovered after 8 weeks of NLC‐TBF 1% gel treatment (*p* = 0.031). *Aspergillus niger* (*n* = 10, 16.6%) was the most frequently isolated NDM species from the nails of patients, followed by *A. terreus* (*n* = 5, 8.3%) and *A. flavus* (*n* = 4, 6.6%). Among the yeast species, *C. tropicalis* (*n* = 8, 13.3%) and *C. albicans* (*n* = 7, 11.6%) were the most prevalent, while *T. mentagrophytes* (*n* = 4, 6.6%) was the sole dermatophyte identified. The TBF‐NLCs demonstrated efficacy against NDMs and TBF‐susceptible strains of *T. mentagrophytes* obtained from patients (*p* < 0.05). However, preliminary AFST against TBF‐resistant *T. mentagrophytes* strains indicated no reduction in the MIC values when using TBF‐NLCs. Consequently, NLCs as a nano‐delivery system were unable to alter the antifungal susceptibility profile of resistant isolates from resistant to susceptible. Consistent with the findings from the preliminary AFST, TBF‐NLCs were ineffective against *Candida* and *Fusarium* species isolated from the patient's nails. It is noteworthy that these isolates exhibited high MIC values against both TBF solution and TBF‐NLCs. Table 6 indicates the molecular characterisation of the causative agents associated with the ONM in this study, along with the outcomes of their AFST. Despite the relative improvement regarding clinical criteria, no significant differences were found among the three groups in aspect of the mycological criteria including KOH‐direct examinations and cultures. During the treatment period (8 weeks), negative results were observed only during the sixth and eighth weeks of TBF‐NLC 1% gel application; however, the *p* values were obtained as > 0.05.

**TABLE 6:** The susceptibility of the fungal species isolated from patients against TBF solution and TBF‐NLCs. The geometric mean, MIC_50_, and MIC_90_ values were also determined.

	TBF‐NLCs				TBF solution			
	MIC	MIC50	MIC90	GM	MIC	MIC50	MIC90	GM
*Aspergillus* sp. (*n* = 28, 46.6%)								
*A. awamori* (*n* = 2, 3.3%)	0.125				0.015			
	0.5				0.08			
*A. amestelodami* (*n* = 3, 5%)	0.0625				0.001			
	0.0625				0.00005			
	0.0625				0.015			
*A. flavus* (*n* = 4, 6.6%)	0.25				0.002			
	0.25				0.002			
	0.5				0.002			
	0.5				0.002			
*A. niger* (*n* = 10, 16.6%)	0.125				0.015			
	0.25				0.08			
	0.125				0.004			
	0.125				0.008			
	0.25				0.004			
	0.125				0.015			
	0.125				0.015			
	0.0625	0.125	0.1641	0.5	0.001	0.0052	0.125	0.004
	0.0625				0.001			
	0.0625				0.001			
*A. versicolor* (*n* = 1, 1.6%)	1				0.3			
*A. sydowii* (*n* = 1, 1.6%)	0.0625				0.002			
*A. terreus* (*n* = 5, 8.3%)	0.0625				0.0002			
	0.5				0.125			
	0.0625				0.0002			
	0.5				0.125			
	1				0.125			
*A. tubingensis* (*n* = 2, 3.3%)	0.25				0.015			
	0.0625				0.0004			
*Fusarium* sp. (*n* = 4, 6.7%)								
*F. proliferatum* (*n* = 4, 6.7%)	4				80			
	2				40			
	2				20			
	4				80			
Dermatophytes (*n* = 4: 6.7%)								
Trichophyton mentagrophytes (*n* = 4: 6.7%)	0.0625				0.002			
	0.0625				0.002			
	0.0625				0.001			
	0.0625				0.001			
*Candida* sp. (*n* = 24, 40%)								
*C. albicans* (*n* = 7, 11.6%)	16				3120			
	0.25				20			
	16				80			
	16				80			
	16				2500			
	16				5000			
	16				5000			
*C. guilliermondii* (*n* = 2, 3.3%)	16				80			
	2				3120			
*C. krusei* (*n* = 1, 1.6%)	16	16	16	5.1873	5000	80	6250	375.24
*C. parapsilosis* (*n* = 6, 10%)	0.5				20			
	0.5				20			
	16				2500			
	16				80			
	1				20			
	2				20			
*C. tropicalis* (*n* = 8, 13.3%)	16				6250			
	0.25				20			
	16				80			
	2				20			
	1				1560			
	16				5000			
	16				6250			
	16				6250			

Abbreviations: GM, Geometric mean; MIC, Minimum inhibitory concentration; MIC50, minimal concentration that inhibits 50% of isolates; MIC90, minimal concentration that inhibits 90% of isolates.

Given that the efficacy of the antifungal drug would be enhanced as an NLC formulation, promising results were obtained for the successful in vitro antifungal effect on both yeast and filamentous fungi. Other studies conducted by the same research team have shown acceptable results for the effect of NLC nanoparticle‐based gel loaded with an herbal agent for the treatment of ONM as well as superficial dermatophytosis [51, 52]. It is worth noting that the findings of Nosratabadi et al. on the positive effects of this combination with new drugs against resistant fungal isolates can be a piece of precise evidence to these results [53]. Recent studies have shown that a lipid carrier‐based nanostructured gel system containing TBF hydrochloride reduces fungal load and this compound has shown promising results in terms of antifungal activity in vivo and can be considered as an effective new formulation in treatment [21, 54, 55, 56, 57, 58, 59, 60]. Surprisingly, the fabricated TBF‐NLCs were most effective against NDM. Hence, it was anticipated that the formulation would be successful when applied to the nail plate. Unfortunately, according to mycological index assessments, no significant improvement was observed even after 8 weeks of TBF‐NLCs 1% gel application. In recent years, many studies have reported on lipid nanoparticles and antifungal drugs. Lipid nanoparticles such as SLN and NLC have shown excellent results in skin penetration. However, only one study using NLC has been published, in which NLC containing voriconazole had a significant effect on the depth of penetration into the thick nail tissue in vitro and a promising response for the management of ONM. However, in contrast to that claim, the surface of the nail plate is such a thick barrier that renders it impervious to a nano‐encapsulated drug. Perhaps other carriers or facilitators are needed to enhance the characteristics of TBF‐nanoparticles to make them penetrable.

## 4 Conclusion

This randomised controlled clinical trial highlighted the promising capabilities of a nanoscale colloidal system in offering safer, more rapid, and effective treatment for ONM. The application of a 1% TBF‐NLCs gel over an eight‐week period resulted in significant satisfaction regarding infection management from both dermatologists and patients. However, no notable adverse outcomes were recorded for both dermatological evaluations and fungal cultures throughout the 8 weeks of TBF‐NLCs treatment. It is strongly suggested that mycological assessments be monitored for more than 8 weeks, with the possibility of extending the follow‐up to 12 weeks. This study presents a promising advancement in topical onychomycosis treatment, demonstrating that TBF‐NLCs achieve rapid clinical and mycological resolution with minimal toxicity. However, this was a small cohort (60 participants), the lack of demographic diversity (e.g., geographic, ethnic) may limit generalisability. Broader validation and long‐term data are essential before this formulation can be widely recommended.

## Author Contributions

**Shima Parsay:** investigation, writing – original draft, data curation, software. **Majid Saeedi:** methodology, supervision. **Mahdi Abastabar:** methodology, investigation. **Mohammad Taghi Hedayati:** methodology. **Seyyed Mobin Rahimnia:** investigation, formal analysis. **Nasim Gholizadeh:** investigation, methodology, resources. **Armaghan Kazeminejad:** investigation, methodology, resources. **Katayoun Morteza‐Semnani:** methodology. **Roozbeh Zare Gashti:** investigation. **Kofi Asare‐Addo:** writing – review and editing, methodology. **Maryam Moazeni:** conceptualization, methodology, data curation, supervision, project administration, funding acquisition, writing – review and editing, resources. **Ali Nokhodchi:** supervision, writing – review and editing, methodology, validation.

## Conflicts of Interest

The authors declare no conflicts of interest.

## Data Availability

The data that support the findings of this study are available from the corresponding author upon reasonable request.
